# Transcriptome analysis reveals the roles of stem nodes in cadmium transport to rice grain

**DOI:** 10.1186/s12864-020-6474-7

**Published:** 2020-02-06

**Authors:** Ailing Liu, Zhibo Zhou, Yake Yi, Guanghui Chen

**Affiliations:** 1 College of Bioscience and Biotechnology, Hunan Agricultural University, Changsha, Hunan 410128 People’s Republic of China; 2 College of Agronomy, Hunan Agricultural University, Changsha, Hunan 410128 People’s Republic of China; 3 Southern Regional Collaborative Innovation Center for Grain and Oil Crops (CICGO), Hunan Agricultural University, Changsha, 410128 People’s Republic of China

**Keywords:** RNA-seq, Cadmium, Panicle node, Node I, Low cadmium accumulation

## Abstract

**Background:**

Node is the central organ of transferring nutrients and ions in plants. Cadmium (Cd) induced crop pollution threatens the food safety. Breeding of low Cd accumulation cultivar is a chance to resolve this universal problem. This study was performed to identify tissue specific genes involved in Cd accumulation in different rice stem nodes. Panicle node and the first node under panicle (node I) were sampled in two rice cultivars: *Xiangwanxian No. 12* (low Cd accumulation cultivar) and *Yuzhenxiang* (high Cd accumulation cultivar). RNA-seq analysis was performed to identify differentially expressed genes (DEGs) and microRNAs.

**Results:**

*Xiangwanxian No. 12* had lower Cd concentration in panicle node, node I and grain compared with *Yuzhenxiang*, and node I had the highest Cd concentration in the two cultivars. RNA seq analysis identified 4535 DEGs and 70 miRNAs between the two cultivars. Most genesrelated to the “transporter activity”, such as *OsIRT1*, *OsNramp5, OsVIT2*, *OsNRT1.5A, and OsABCC1*, play roles in blocking the upward transport of Cd. Among the genes related to “response to stimulus”, we identified *OsHSP70* and *OsHSFA2d/B2c* in *Xiangwanxian No. 12*, but not in *Yuzhenxiang*, were all down-regulated by Cd stimulus. The up-regulation of miRNAs (*osa-miR528* and *osa-miR408*) in *Xiangwanxian No. 12* played a potent role in lowering Cd accumulation via down regulating the expression of candidate genes, such as *bZIP*, *ERF*, *MYB*, *SnRK1* and *HSPs*.

**Conclusions:**

Both panicle node and node I of *Xiangwanxian No. 12* played a key role in blocking the upward transportation of Cd, while node I played a critical role in *Yuzhenxiang*. Distinct expression patterns of various transporter genes such as *OsNRT1.5A, OsNramp5, OsIRT1, OsVIT2* and *OsABCC1* resulted in differential Cd accumulation in different nodes. Likewise, distinct expression patterns of these transporter genes are likely responsible for the low Cd accumulation in *Xiangwanxian No. 12* cultivar*.* MiRNAs drove multiple transcription factors, such as *OsbZIPs, OsERFs, OsMYBs*, to play a role in Cd stress response.

## Background

Rice (*Oryza sativa*) is one of the largest food crops in China, accounting for 60% of the basic food supply. In recent years, an increasing area of rice fields in China has been contaminated by heavy metal cadmium (Cd). In the 2010s, the reduction of annual grain production by heavy metal pollution is about 100 billion tons [1]. Cd pollution has caused an irreversible and difficult problem in rice production in China, especially in the southern regions. Physical, chemical, and phytoremediation strategies have been widely used to treat Cd-contaminated soils, but little was recovered due to the high technical difficulties or costs. Therefore, it remains an urgent issue in solving the problem of Cd pollution.

Plants have evolved a plethora of genetic and metabolic mechanisms against Cd stresses. The Cd accumulation capacity in different rice varieties varies greatly [2, 3]. One of the possible solutions for alleviating Cd contamination in rice is to cultivate varieties with less Cd accumulation in grains. The use of molecular and transgenic technologies coupled with next-generation sequencing (NGS) could facilitate the identification of genes and mechanisms potentially involved in the translocation, detoxification, immobilization, and allocation of Cd in different species and cultivars [4–7]. Recently, some genes involved in Cd uptake, transport and accumulation had been identified and used as targets of genetic manipulation [8–10]. Zhang et al. (2010) found that the *BanCn.ABCC3* played a pivotal role in Cd resistance in rapeseed by blocking Cd transport to seeds and retaining Cd in the root pectin and shoot vacuoles [5]. Luo et al. (year) found that the loss-of-function mutation of Arabidopsis *PLANT DEFENSIN 2* (*AtPDF2.5*) reduced Cd accumulation and enhanced Cd resistance in Arabidopsis root by chelating Cd [4]. Besides, *CAL1* also plays a role in Cd transport by chelating Cd ion in the cytoplasm and facilitating Cd secretion to extracellular space [11]. However, the limited number of genes is insufficient to fully understand the biological processes of Cd transport and accumulation in plants.

Studies have shown that node is a pivotal location for nutrient distribution in graminaceous plants [12]. Both root and node are key barriers to Cd transport into rice grains [13]. Node is a central organ for xylem-to-phloem transfer of nutrients, ions, and metabolites [12]. Stem nodes play a vital role in Cd transfer from soil to grains [14]. Genetic manipulation of the transporters in stem-portion might prevent the distribution of toxic heavy metals, like Cd, into grains [15]. Feng et al. (2017) reported that the Cd concentration profiles were distinct in different part of rice, including stem nodes [13]. They showed that node I had higher capacity in Cd sequestration and detoxification, and node I had higher expression of genes associated with glycolysis and detoxification. Fujimaki et al. (2010) found that Cd accumulated most intensively in rice nodes [14]. These results indicate the multifaceted roles of plant nodes in Cd accumulation and detoxification. However, little is known about how differentially expressed genes (DEGs) related to Cd transport and enrichment of Cd in rice nodes.

Rice variety “Xiangwanxian No. 12” with low Cd-accumulation and “Yuzhenxiang” with high Cd accumulation in the grains were identified in previous study [16]. In this study, we performed deep sequencing analysis to identify DEGs and miRNAs (DEmiRNAs) between node I and panicle node from the two cultivars with and without-Cd stress. Through bioinformatics analysis, the key candidate genes, miRNAs, and biological processes in response to Cd stress were deciphered. These results are useful in the future elucidation of the molecular mechanisms of Cd-accumulation and transport to rice grains.

## Results

### Cd accumulation during cd-stress

**Fig. 1:**
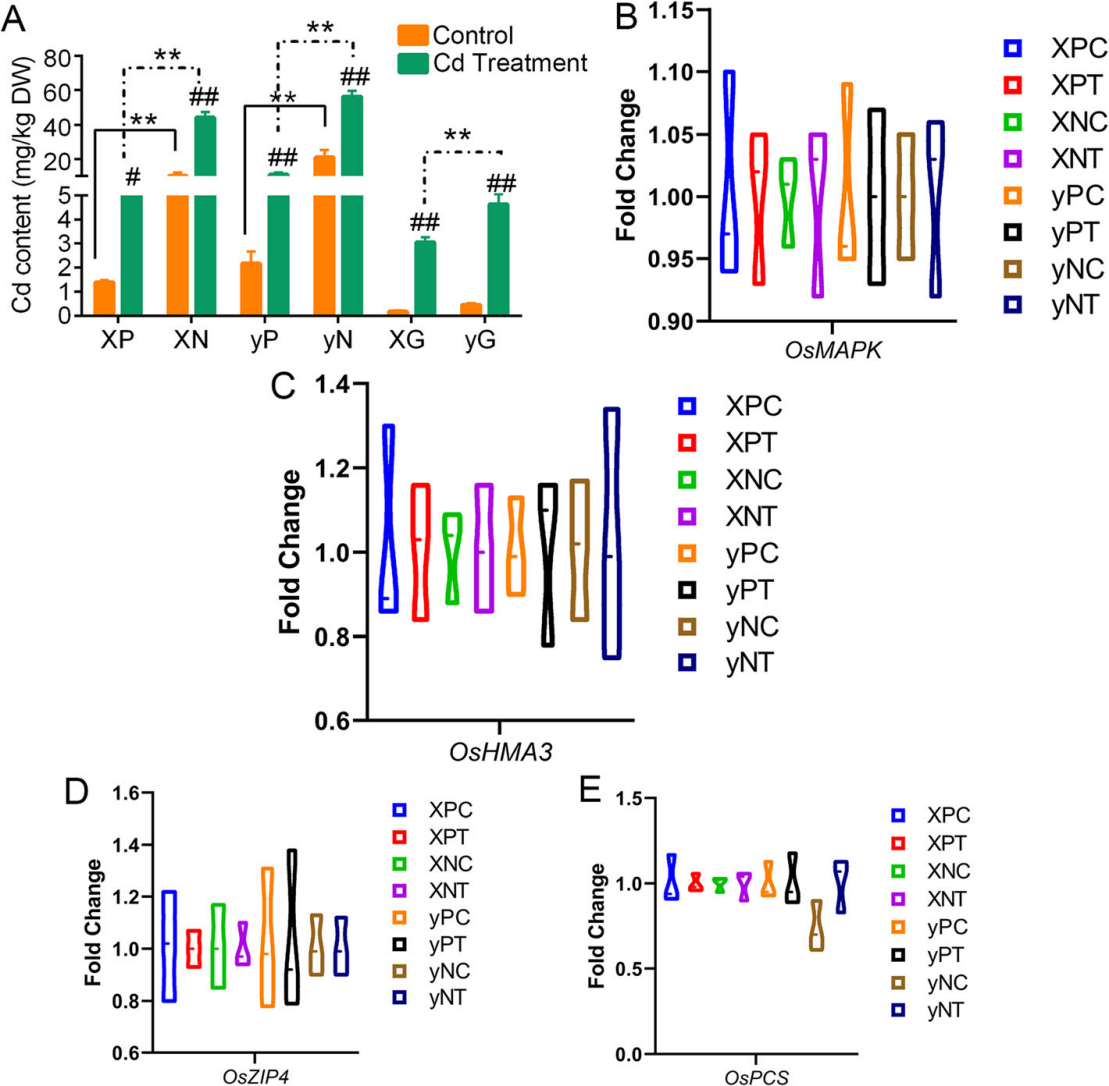
Cd contents and regulator expression profiles in these two rice varieties. **a** shows the Cd contents in different stem nodes and grain. DW, dry weight (kg); XP, panicle node of *Xiangwanxian No.12*; XN, node I of *Xiangwanxian No.12*; yP, panicle node of *Yuzhenxiang*; yN, node I of *Yuzhenxiang*; XG, grain of *Xiangwanxian No.12*; yG, grain of *Yuzhenxiang*. # *p* < 0.05, ## *p* < 0.01 Cd treatment vs. control; * *p* < 0.01, ** *p* < 0.05 y (*Yuzhenxiang*) vs. X (*Xiangwanxian No.12*). **b** to **e** show the interleaved violin plot of the expression of *OsMAPK*, *OsHMA3*, *OsZIP4* and *OsPCS*, respectively

Under the control condition, node I (marked as “N”) in the two cultivars [21.05 mg/kg DW (Dry Weight) in “Yuzhenxiang” as “y” and 10.25 mg/kg DW in “Xiangwanxian No. 12” as “X”] had higher Cd accumulation compared with panicle node (marked as “P”, 2.17 mg/kg DW in “y” and 1.40 mg/kg DW in “X”; *p* < 0.01, Fig. 1a). The Cd stress increased the Cd accumulation in all tissues, especially in the node I (56.43 mg/kg DW in “y” and 44.25 mg/kg DW in “X”). Grains of “X” cultivar had lower Cd content (both in control and Cd treatment) than that in “y” cultivar. These data confirmed that “y” was a high Cd accumulation cultivar, and node I had higher capacity in Cd sequestration. In addition, the expression of *OsMAPK, OsHMA3, OsZIP4* and *OsPCS* showed different profiles in different groups (Fig. 1b to e). *OsMAPK* showed a higher expression (mean value) after Cd treatment in panicle node and node I of “X” and “y”. Additionally, the expression of *OsMAPK* in “X” was higher than that in “y” (Fig. 1b). We found that the expression of *OsHMA3* was increased by Cd stress in panicle node, not in node I (Fig. 1c). While *OsZIP4* and *OsPCS* showed no differences among different groups (Fig. 1d and e).

### Summary of the mRNA-seq and miRNA-seq

**Fig. 2:**
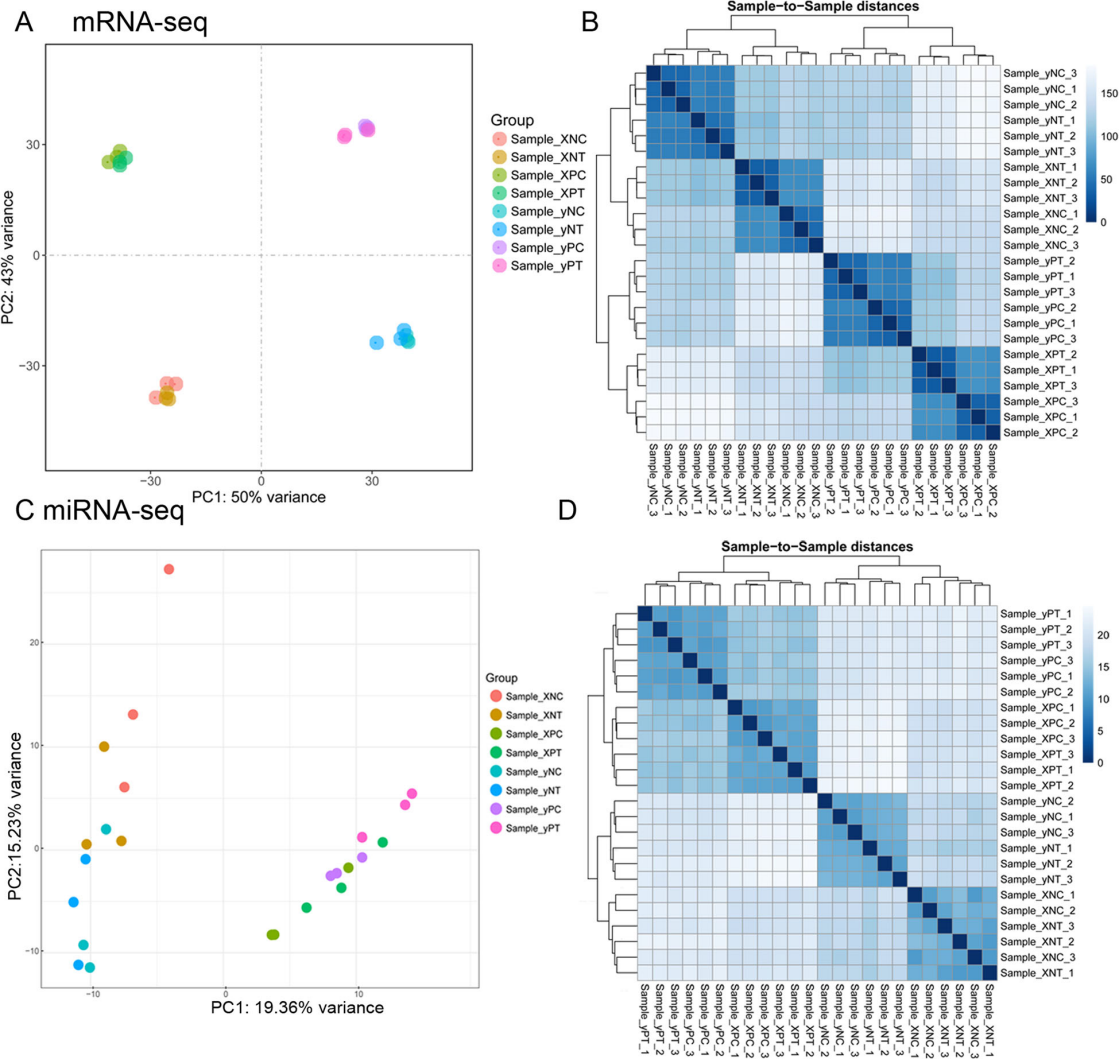
Sample clustering and correlation analysis. **a** and **c** the principal component analysis (PCA) of the samples sequenced using the mRNA and miRNA expression level, respectively. **b** and **d** the sample-to-sample clustering analysis based on the mRNA and miRNA expression level, respectively. The color depth notes the similarity between samples (0~1). The deeper the color, the higher the similarity

Twenty-four cDNA libraries were constructed and a total of 1111.34 M clean reads obtained with 89.80% average mapping rate (76.45–92.37%) to the rice reference genome (Additional file 2: Table S1). Principle component analysis (PCA) (Fig. 2a) and sample-to-sample clustering analysis (Fig. 2b) showed that the samples of the same tissue (“P” or “N”) from the same cultivar of control (“C”) and Cd treatment (“T”) were clustered together, respectively. Twenty-four miRNA libraries generated total 350.83 M clean reads (112.57 M unique reads) with an averaged mapping rate of 82.01% to the *O. sativa* reference miRNA in miRbase (http://www.mirbase.org/cgi-bin/mirna_summary.pl?org=osa). The alignment rate of each sample ranged from 0.62 to 2.37% in miRBase (average 1.49%, Additional file 2: Table S1). PCA and sample-to-sample clustering analysis showed that samples of the same tissue (“P” or “N”) were grouped together (Fig. 2c and d).

### DEGs between high and low cd accumulation cultivars

**Fig. 3:**
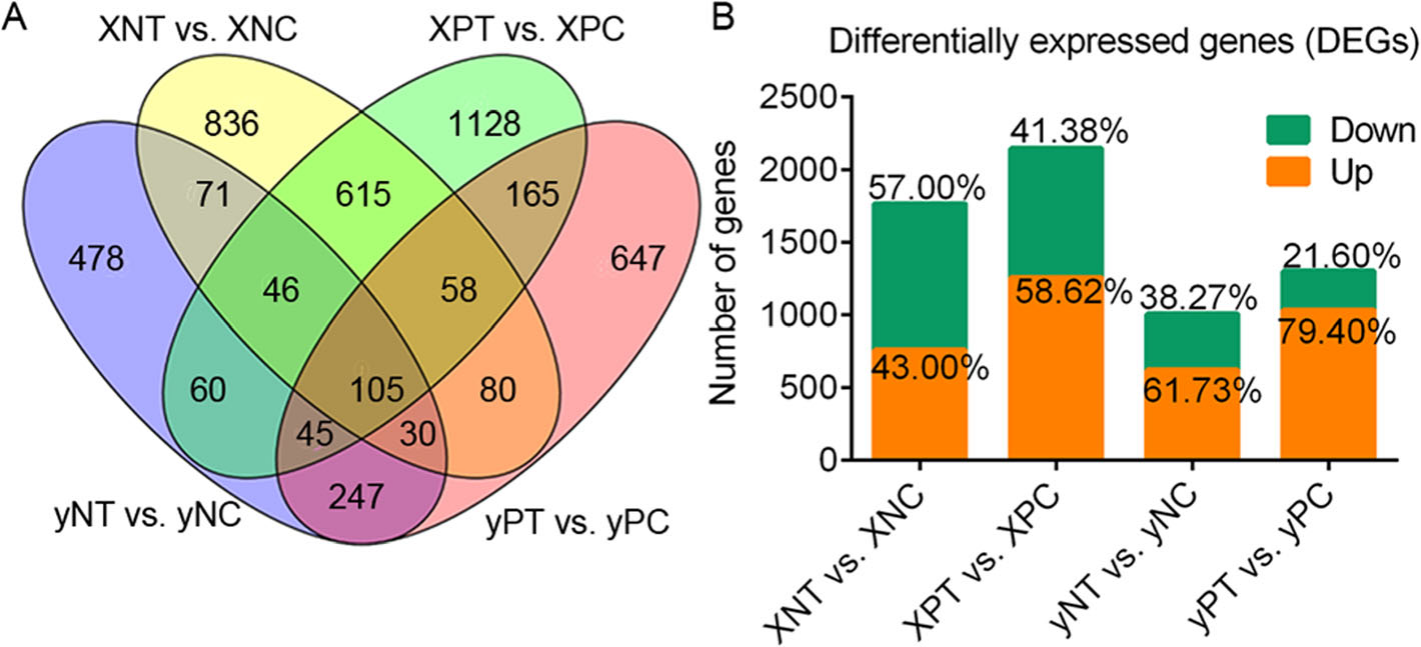
Summary of differentially expressed genes (DEGs). **a** The Venn figures of the DEGs in pairwise comparison of Cd-treated (T) vs. untreated control (C) of node I (N) and panicle node (P) in “X” and “y” cultivar, respectively. **b** the statistics of the up- and down-regulated DEGs. The number on the column indicates the percent of the up and down-regulated DEGs by each comparison

**Fig. 4:**
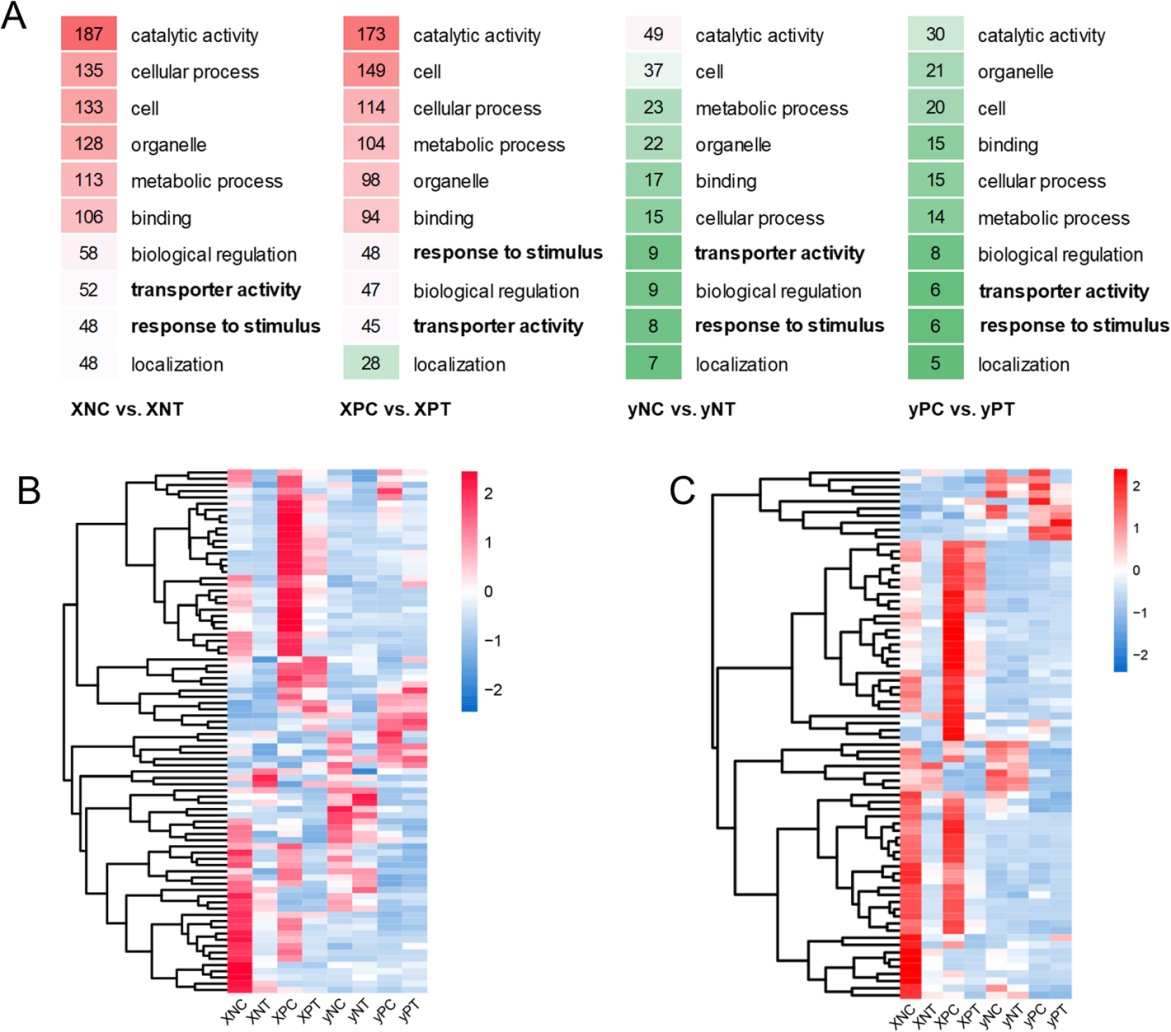
Differentially expressed genes (DEGs) between high and low Cd accumulation cultivars. **a** Gene Ontology (GO) and KEGG pathway enrichment analyses of down-regulated DEGs in pairwise comparison of Cd-treated (T) vs. untreated control (C) of node I (N) and panicle node (P) in “X” and “y” cultivar, respectively. The redder the color, the more significantly enriched DEGs there were, the greener the color, the less; **b** and **c** Hierarchical clustering analysis of the DEGs involved in “transporter activity” (B) and “response to stimulus” (C), respectively

A total of 4535 DEGs were identified by pairwise comparison of Cd-treated (T) vs. untreated control (C) of node I (N) and panicle node (P) in “X” and “y” cultivar, respectively (Fig. 3a-b and Table 1). The results showed that there were more down-regulated genes in “X” cultivar than that in “y” cultivar. GO and KEGG enrichment based on these down regulated genes were performed to identify the main biological processes. The results showed that GO terms of “transporter activity” and “response to stimulus” were significantly enriched in “X”, not in “y” (Fig. 4a). Results of hierarchical clustering analysis of the 84 common DEGs (including 69 genes down regulated by Cd stress in “X”) showed an enrichment in “transporter activity” (Fig. 4b and Additional file 3: Table S2), whereas another 74 common DEGs (including 62 genes down regulated by Cd treatment in “X”) were enriched in “response to stimulus” (Fig. 4c and Additional file 4: Table S3). Notably, most of the DEGs in “X” were down-regulated by Cd treatment, and most down-regulated genes in “X” by Cd treatment were unchanged in “y”, especially for the DEGs associated with “response to stimulus” (Fig. 4c). Most of the DEGs mentioned above were differently expressed in different stem nodes (Fig. 4c and d).

**Table 1:** Statistics of the differentially expressed genes (DEGs) and miRNAs (DEmiRNAs) by different pairwise comparison

Comparison		DEG			DEmiRNAs		
Case	Control	Up	Down	Total	Up	Down	Total
XNT	XNC	759	1006	1765	20	10	30
XPT	XPC	1258	888	2146	11	14	25
yNT	yNC	621	385	1006	15	10	25
yPT	yPC	1033	268	1301	18	8	26

“X” notes low Cd accumulation cultivar “Xiangwanxian No. 12”, “y” notes high Cd accumulation cultivar “Yuzhenxiang”, “P” indicates panicle node, “N” indicates the first node, “C” represents control and “T” represents Cd treatment

The iron-regulated transporter 1 (*IRT1*, OS03G0667500) [17] and metal transporter Nramp5 (Mn and Cd uptake protein, OS07G0257200) [18–20] were noteworthy as they had higher expression in the panicle node compared with node I (Additional file 3: Table S2). For node I, *OsIRT1* and *OsNramp5* increased in “X”, while decreased in “y” cultivar (Additional file 1: Figure S1 A). Although the expression of *OsIRT1* and *OsNramp5* increased after Cd treatment, overall it remained at a low level in “X” cultivar.

The expression patterns of *OsNRT1.5A* (OS02G0689900, nitrate transporter 1.5A) and *OsVIT2* (OS09G0396900, Vacuolar Iron Transporter 2) implied its key role in the upward transport of Cd [21, 22]. *OsNRT1.5A* had a higher expression level in node I than that in panicle node in both two cultivars. In the panicle node, *OsNRT1.5A* was down-regulated in “X” but up-regulated in “y” under Cd stress. *OsVIT2* was up-regulated following Cd treatment in node I in both cultivars, but down-regulated in panicle node in the “X” cultivar. Cd stress had little effects on the expression level of *OsVIT2* in panicle node in “y” cultivar (Additional file 1: Figure S1 A and Additional file 3: Table S2). Aquaporin protein is closely related to heavy metal stress, with distinct expression patterns in different plant species [23–27]. In our results, 6 aquaporin genes (PIPs) (OS02G0629200, *OsPIP2–6*; OS04G0233400, *OsPIP2–6*; OS02G0666200, *OsPIP1–1;* OS04G0559700, *OsPIP1–2*, OS07G0448100, *OsPIP2–4*; OS07G0448800, *OsPIP2–1*) showed similar expression profiles under Cd stress (Additional file 1: Figure S1 B). The expression of 6 *PIPs* members was higher in “X” than in “y” cultivar under control condition, and down regulated after Cd treatment in “X”, but with little changes in “y”. The expression of *OsPIPs* show no significant differences between node I and panicle node in “X” and “y”.

Among the DEGs related to “response to stimulus”, heat shock transcription factor (*HSF*) *A2d/B2c* genes (including OS03G0161900/OS09G0526600), light-harvesting chlorophyll a-b binding protein (*LHC-II*) genes (e.g. OS02G0197600), and genes encoding heat shock protein (*HSP71.1/70/20*; including OS03G0276500, OS01G0840100 and OS06G0253100) showed similar changes in the panicle node and node I of the two rice cultivars (Additional file 4: Table S3). Higher expression levels of aforementioned genes were found in “X” than in “y” cultivar (Additional file 1: Figure S1C). Cd treatment decreased the expression of all these genes in the “X” cultivar, but increased *OsHSF*-A2d/B2c, *OsLHC-II* and *OsHSP71.1* in the panicle node of “y” cultivar. The distinct expression profiles of these DEGs mentioned above are likely account for, or in part, the differential Cd accumulation between the two rice cultivars.

### Expression of known cd-responsive genes

**Fig. 5:**
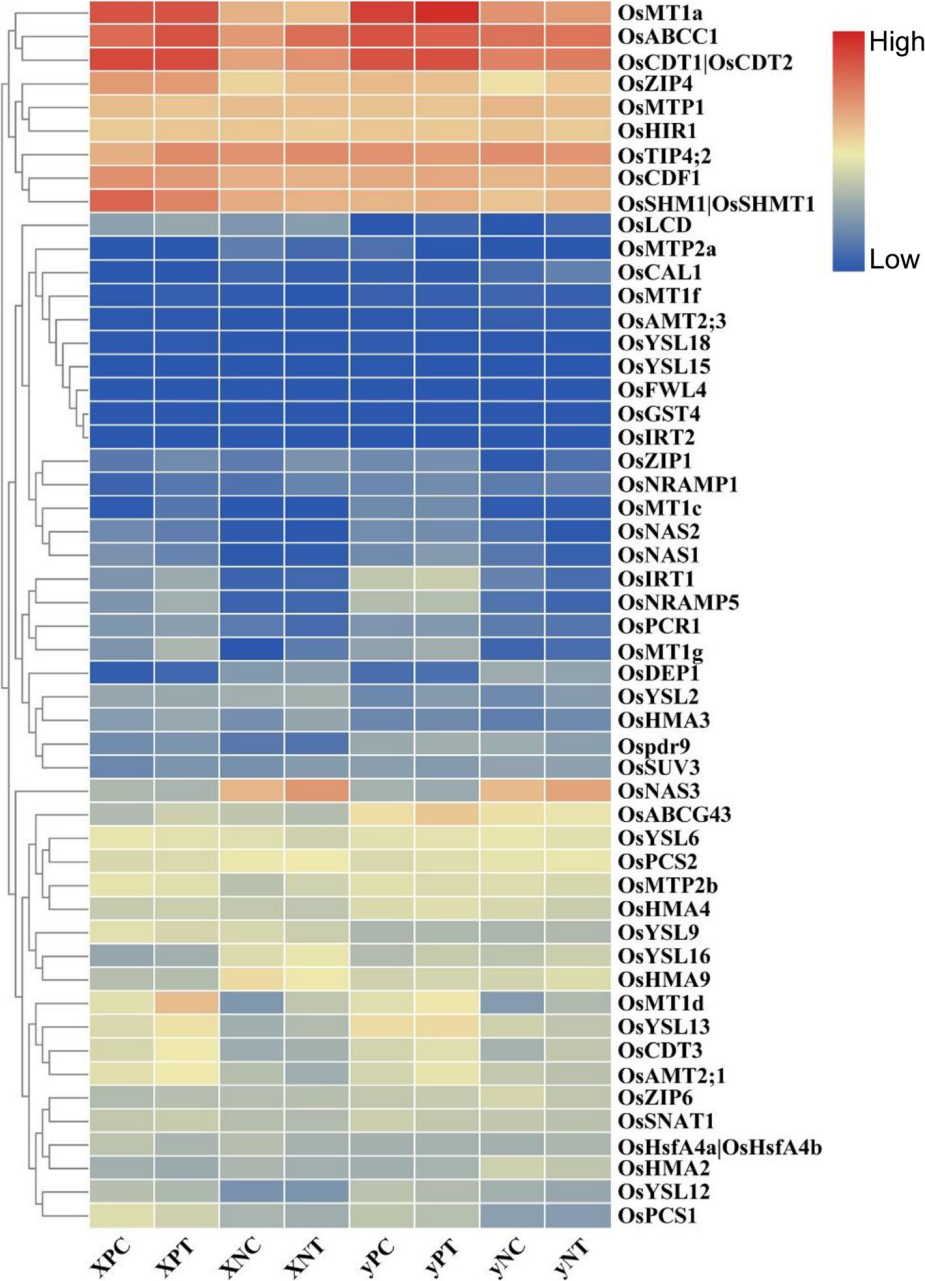
Heatmap of known 52 Cd-responsive genes. These Cd-responsive genes were collected from previous literatures. The change of color from blue to red indicated that gene expression level was low to high

In order to decipher the DEGs expression pattern in different stem nodes under Cd stress, we analyzed the expression profiles of 52 Cd-responsive genes reported previously in the literature. Among these genes, metallothionein 1 (*OsMT1*) [28], cadmium tolerant 1 (*OsCDT1*), *OsCDT2* [29, 30], *OsMTP1* [7], cation diffusion facilitator (*OsCDF1*), ATP-binding cassette transporter multidrug resistance protein 1 (*OsMRP1*/*ABCC1*) [31, 32] showed higher expression levels in the two rice cultivars. Furthermore, they expressed higher in the panicle node of the two cultivars compared with node I (Fig. 5). Cd stress enhanced the expression of *OsCDT1* and *OsABCC1* in the node I of “X” cultivar. Another gene serine hydroxymethyltransferase 1 (*OsSHM1*), which showed relatively higher expression level, was down regulated only in the panicle node of “X” cultivar by Cd stimulus (Fig. 5). In addition, the *OsNAS3* (nicotinamine synthase 3) and *OsDEP1* (dense and erect panicle 1) showed relatively high expression in node I of the two cultivars. Cd treatment increased the expression of *OsNAS3* and *OsMT1d* in node I and both nodes, respectively (Fig. 5). Other Cd-responsive genes including *OsMT1f, OsYSL15, OsIRT2,* and *OsGST4* had low expression levels in the two cultivars, which were unresponsive to Cd stress in our study.

### Identification of differentially expressed DEmiRNAs

**Fig. 6:**
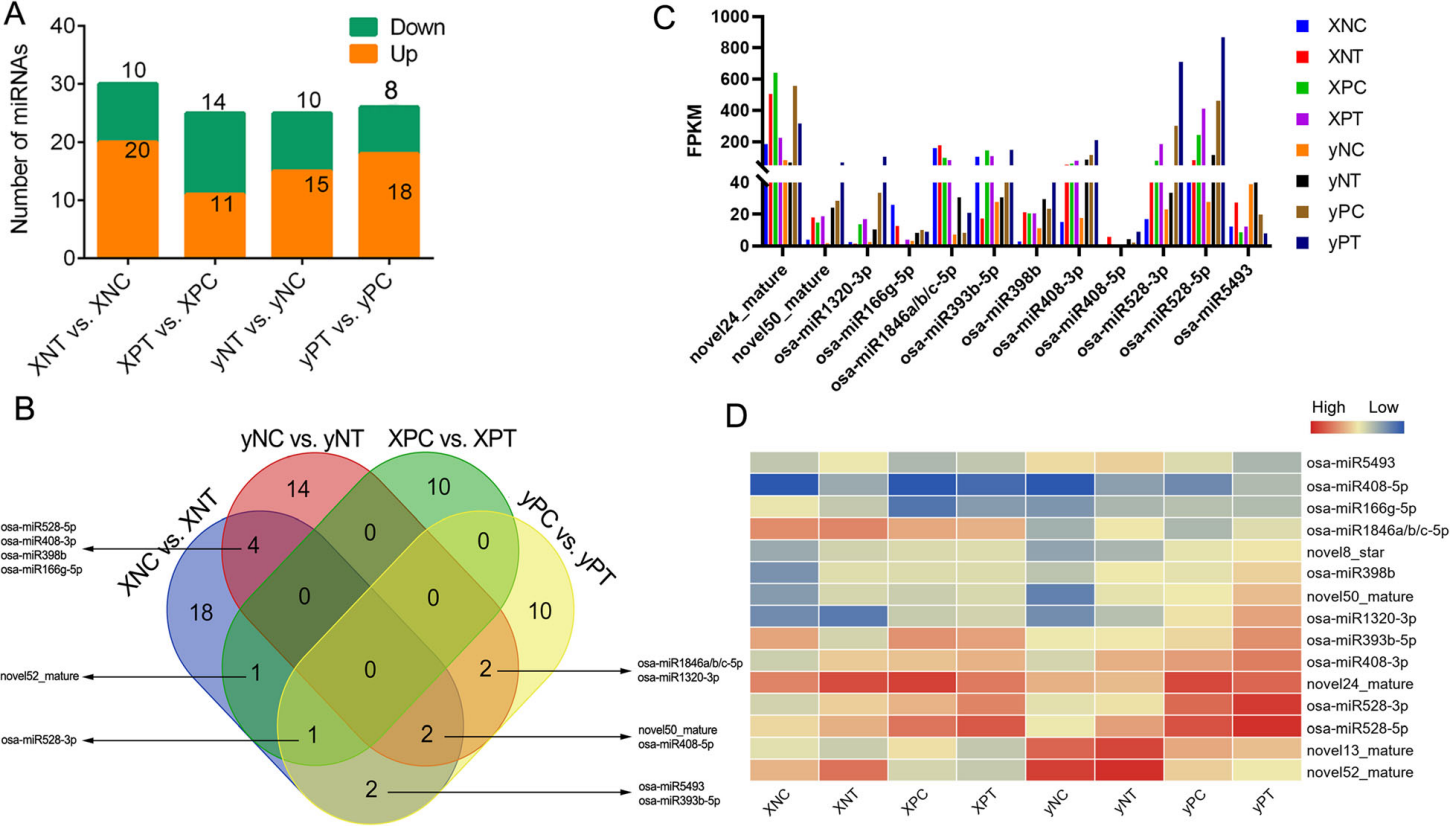
Differentially expressed miRNAs (DEmiRNAs) responsible for low Cd accumulation. **a** The statistics of the up- and down-regulated DEmiRNAs. The number on the column indicates the number of the up and down-regulated DEGs by each comparison; **b** The Venn figures of the of DEmiRNAs in Node I DEGs in pairwise comparison. The common DEmiRNAs were pointed out by the arrow; **c** Expression of the common DEmiRNAs in Cd-treated (T) vs. untreated control (C) of node I (N) and panicle node (P) in “X” and “y” cultivar, respectively; **d** Heatmap of the candidate miRNAs and their differentially expressed family members. The change of color from blue to red indicated that gene expression level was low to high

**Fig. 7:**
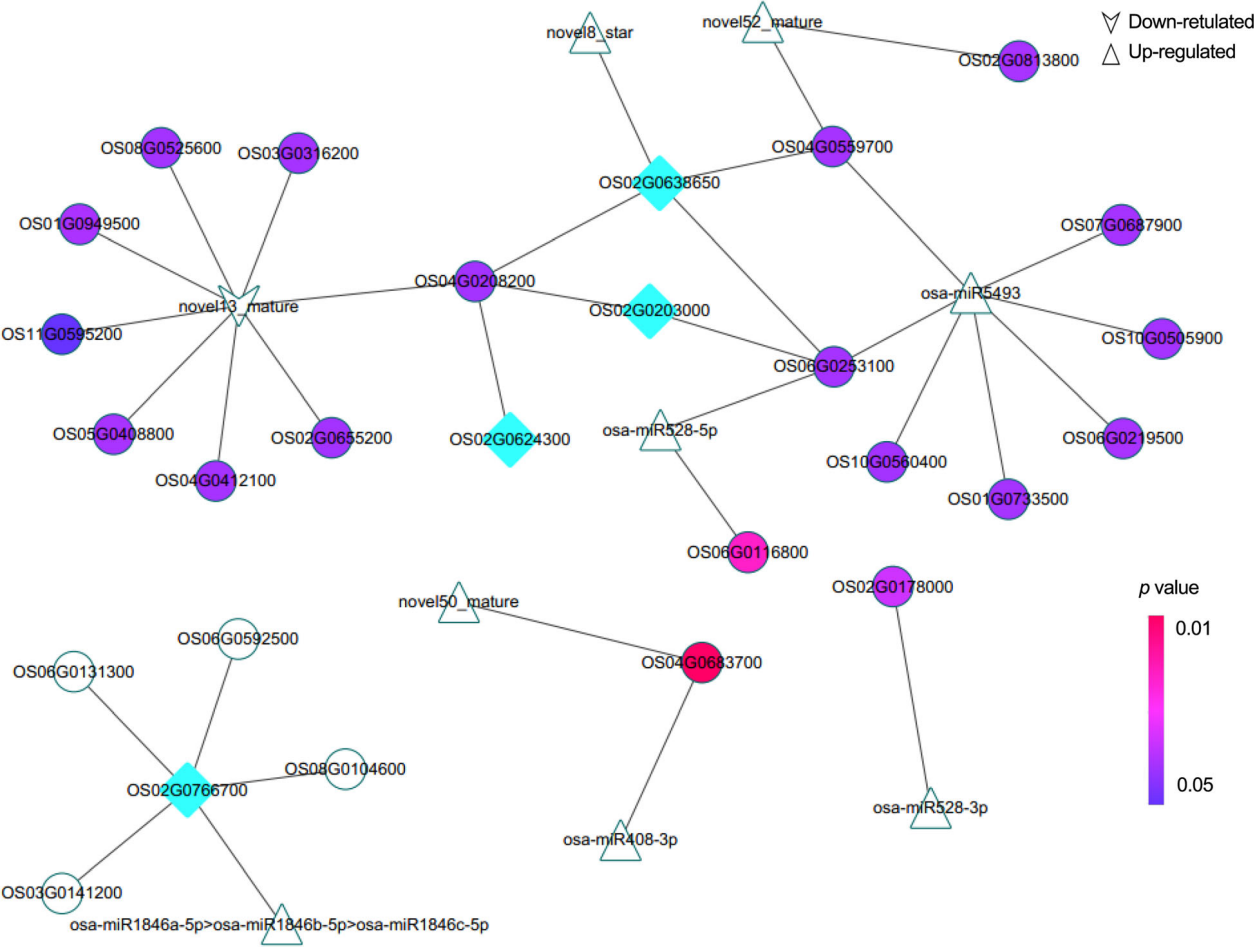
Regulatory network of candidate miRNAs and target genes.  represented down-regulated differentially expressed miRNAs (DEmiRNAs) after Cd teratment;  represented up-regulated DEmiRNAs after Cd treatment;  represented the target genes. Different *p* values are expressed in different colors, as shown in the figure.  indicated that the target gene was a transcription factor. Different *p* values are expressed in different colors, as shown in the figure

A total of 70 non-overlapping DEmiRNAs were identified from panicle node and node I in the two rice cultivars (Fig. 6a and Additional file 5: Table S4). Most DEmiRNAs were up-regulated by Cd treatment. There were 12 common DEmiRNAs among all the pairwise comparisons (Fig. 6b), of which six miRNAs (*osa-miR398b*, osa*-miR408-3p*, osa*-miR408-5p*, osa-*miR528-5p, osa-miR528-3p and novel50_mature*) were up-regulated by Cd treatment (Fig. 6c). In addition, only one common DEmiRNA (*osa-miR528-3p*) was identified as Cd up-regulated in the panicle node of both cultivars. There were six common DEmiRNAs (osa*-miR408-3p*, osa*-miR408-5p*, osa-*miR528-5p, osa-miR398b, osa-miR166-5p* and *novel50_mature*) in node I of both cultivars, among which, only *osa-miR166-5p* showed a different expression pattern in node I of “X” cultivar and panicle node of “y” cultivar. In order to reveal the differential expression profiles of microRNAs more comprehensively, we screened the other miRNA family members (Fig. 6d). O*sa-miR398b*, osa*-miR408-3p*, osa-*miR528-3p* and osa-*miR528-5p* had a higher expression level in panicle node than that of node I under Cd stress. We then constructed the DE miRNA-mRNA regulatory network (Fig. 7) based on the 15 miRNA (12 common and 3 family members) and the targets among the down-regulated DEGs. In the regulatory network, an *HSP* member (OS06G0253100) was regulated by two miRNAs including *osa-miR528-5p* and *osa-miR5493*. *OsMYB5P* (OS02G0624300), *OsbZIP18* (Os02g0203000), and *OsERF141* (Os02g0638650) were regulated by *osa-miR528-5p* and novel13_mature, indirectly. Another bZIP member *OsbZIP23* (Os02g0766700) was the target gene of *osa-miR1846a/b/c-5p*; SNF1-related protein kinase 1 subfamily protein (SnRK) gene (OS02G0178000, *OsSnRK1*) was regulated by *osa-miR528-3p*. In addition, *OsAAE3* (OS04G0683700) was regulated by both *osa-miR408-3p* and *novel50_mature* (Fig. 7 and Additional file 1: Figure S1D).

### qRT-PCR verification of RNA-seq data

**Fig. 8:**
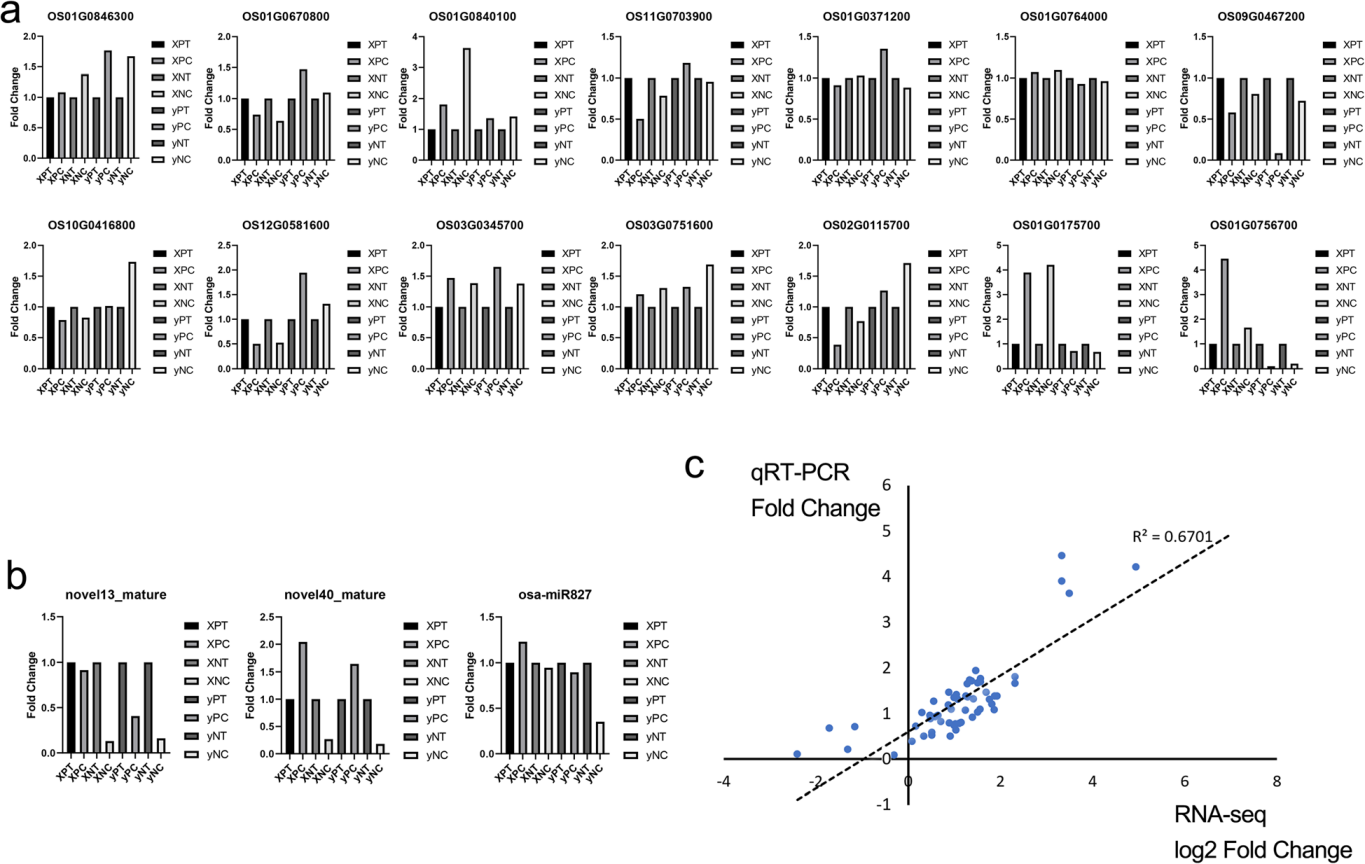
Consistency results of qRT-PCR and RNA-seq. **a** Expression of mRNAs in different group. **b** Expression of miRNAs in different group. **c** Consistency results of qRT-PCR and RNA-seq (including mRNAs and miRNAs). The figure was based on the fold change of qRT-PCR and log2 fold change of RNA-seq. R^2^ represented the correlation coefficient between qRT-PCR and RNA-seq

A total of 14 mRNA and 3 miRNAs were selected randomly for qRT-PCR analysis (Fig. 8a and c). The fold-changes of all the selected 14 mRNA and 3 miRNAs found in qRT-PCR and RNA-seq were highly consistent, and the correlation coefficient was 0.6 (Fig. 8c).

## Discussion

Cd has serious influences on photosynthesis [33, 34], respiration [35], nutrient metabolism, distribution and ion transport in plants [8, 36–38]. Identification of rice cultivars with low Cd accumulation in the grains is of highly theoretical and practical significant in rice breeding. Our present study confirmed that the node I of rice plant had a high capacity in Cd sequestration and accumulation. Results of our RNA-seq analyses indicated that different capacities in Cd accumulation between node I and panicle node were mediated by different gene expression pattern in different rice cultivars.

### Barring cd transport into rice grains

The high Cd accumulation in the nodes and roots of rice has been reported by Feng et al. [13]. Node is the central organ of xylem to phloem transport of nutrients, ions, and metabolites [12]. It plays a vital role in Cd transport from soil to grains [14]. Previous reports showed that the accumulation of heavy metals gradually decreased in successive nodes [13, 39–41]. Cd is transported upward and accumulated in nodes, distributed in the xylem elliptical vascular bundles and the surrounding parenchyma cell bridges [14, 39]. In the present study, the accumulation of Cd in nodes is obvious, consistent with a previous report [14]. But the content of Cd in different nodes is different, so the roles of different nodes in blocking the upward transport of Cd are distinct. The high Cd content in node I indicated that most Cd was blocked here during upward transport. The Cd transport was subsequently blocked in panicle node, although to a lesser extent. Therefore, it appears that the upward transport of Cd decreases step by step from node I to panicle node, so the concerted effect of the two nodes is important for the interception of Cd in rice stem.

### Key genes mediating the cd transport and accumulation in rice node I and panicle node

Using the transcriptome data, we identified several key genes might be responsible for the Cd accumulation in node I and panicle node. For panicle node, compared with the high Cd-accumulation cultivar “y”, low Cd-accumulation cultivar “X” had lower expression of *OsIRT1* and *OsNramp5*, but higher expression of *OsVIT2* and *OsNRT1.5A*. Nramp5 is an Mg and Cd transporter as well as Mn and Cd uptake protein. The uptake of Cd into the root cells is primarily mediated by *OsNramp5*, which showed higher transport activity than its counterpart in wheat or maize [18–20]. The knockout or loss-of-function mutation of *OsNramp5* dramatically reduced the accumulation of Cd and Mn without compromising yield [18, 42]. Compared with *OsNramp5, OsIRT1* has a relatively small contribution to Cd uptake [17]. Cd stress reduced the expression of *OsNramp5* and *OsIRT1* in node I of “y”, which indicated that the Cd intake capacity faded. As for “X”, *OsNramp5* and *OsIRT1* were up-regulated in both node I and panicle node after Cd treatment, of which the expression still at a low level, although they were increased. This indicated that the response patterns of *OsNramp5* and *OsIRT1* to Cd stress were distinct between “X” and “y”, especially in node I. It is likely that “X” cultivar reduces Cd intake by maintaining a low expression level of *OsNramp5* and *OsIRT1* in both node I and panicle node, while “y” cultivar blocked Cd uptake mainly by reducing *OsNramp5* expression in node I.

*VIT2* regulates metal sequestration into vacuoles. *VIT2* is up-regulated when excessive metals are available in the environment. The enhanced *VIT2* expression consequently leads to higher vacuolar sequestration capacity and metal accumulation in vacuoles [43–45]. Similar to *OsVIT2, OsABCC1* can concentrate heavy metal ions in vacuoles and prevent it from upward transport to the grains [5, 32, 46]. Our transcriptome data showed that *OsVIT2* was up- and down-regulated in node I and panicle node in “X” cultivar, respectively. As for “y”, there were little changes in the expression of *OsVIT2.* In particular, the expression of *OsVIT2* in node I of “y” maintained a higher level than “X”, which might explain the high content of Cd in “yN”. In addition, the expression of *OsABCC1* was induced by Cd treatment in “X”, but not in “y” cultivar (Fig. 5). The enhanced expression of *OsABCC1* might contribute to the relatively low Cd accumulation in grains of “X” cultivar.

*NRT1.5A* mediated nitrate distribution plays a role in plant tolerance to Cd stress [47]. The NO_3_^−^negatively affects Cd uptake in plant roots [48, 49]. In our results, the expression pattern of *OsNRT1.5A* was completely different in different nodes. *OsNRT1.5A* in node I was several times higher than that in panicle node, which implicated that *OsNRT1.5A* mainly played a role in node I. Cd treatment enhanced *OsNRT1.5A* expression in node I in “X” cultivar, but not in “y” cultivar, which revealed a differential response in the two rice cultivars. Because NRT1 negatively regulates the uptake of Cd and other cations by simultaneously uptake of NO_3_^−^ in Arabidopsis [48], our results could be explained by the same inference, as high expression of *OsNRT1.5A* would suppress Cd uptake in node I in “X” cultivar.

**Fig. 9:**
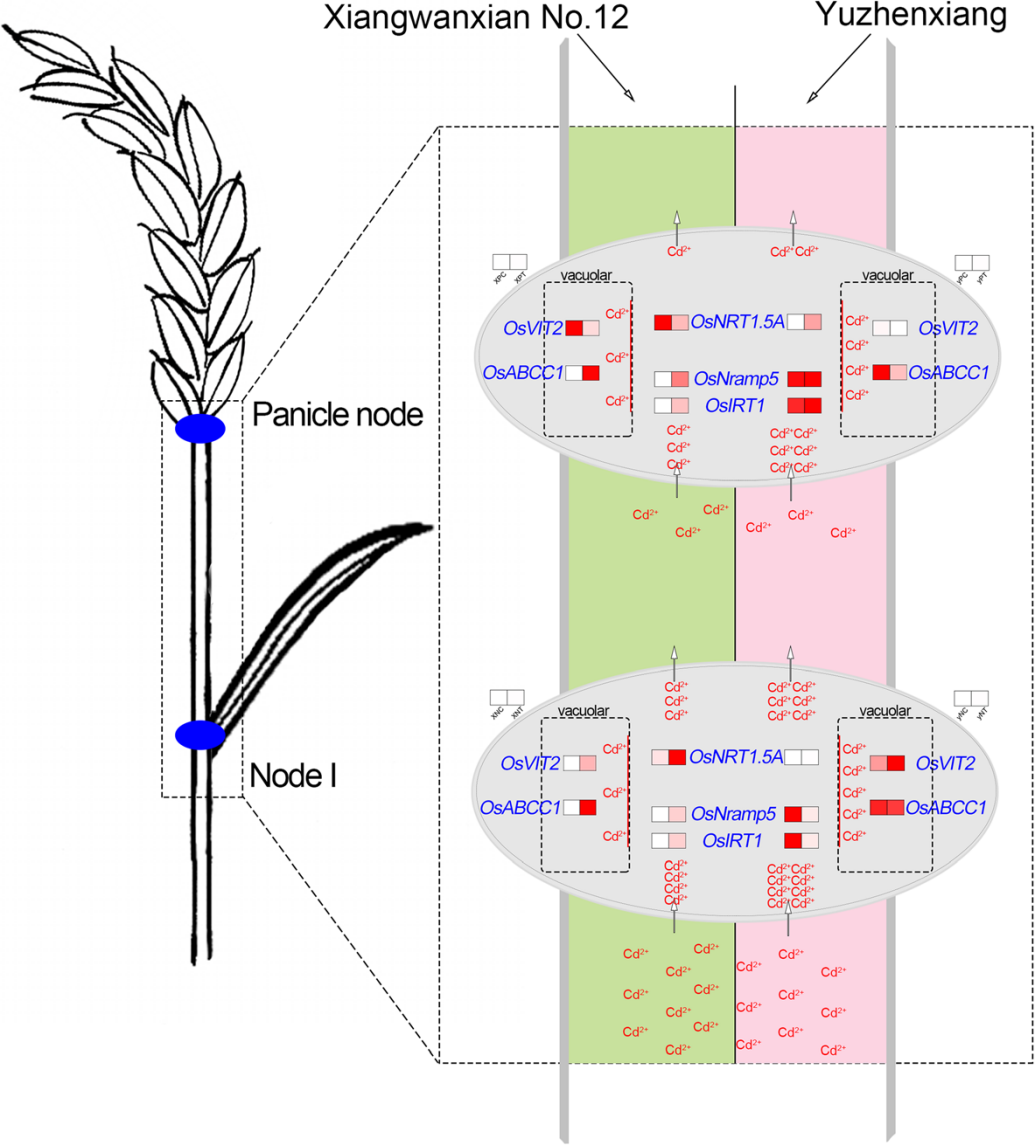
Schematic maps of upward transport mechanism of blocking Cd in different rice varieties. Panicle node (P) and node I (N) were marked in blue on the rice sketch. The schematic map (the right of the figure) showed the upward transport mechanism of blocking Cd in *Xiangwanxian No.12* (pale green) and *Yuzhenxiang* (pale pink). The ellipse at top and bottom represented panicle node and node I, respectively. The expression of key genes was showed by heatmap (box on left and right represented control and Cd treatment, respectively), the redder the color, the higher the expression. The direction of Cd transportation was indicated by arrows and the content of Cd was expressed by the number of “Cd^2+^”

The concerted expression of aforementioned genes is likely to have a large impact on Cd accumulation in rice nodes and grains. The proposed mechanism is shown in Fig. 9.

### Key genes responded to the cd stimulus in node I and panicle node

Among the DEGs related to “response to stimulus”, *OsHSFA2d/B2c, OsLHC-II*, and *OsHSPs* (including *OsHSP70* and *OsHSP20.0*) showed higher expression in the low Cd-accumulation “X” compared with high Cd-accumulation “y” cultivar (Additional file 4: Table S3, Additional file 1: Figure S1). The expression of all these genes was significantly reduced by Cd treatment in “X”, but showed small or undetectable changes in “y” cultivar, indicating a relative insensitivity of “y” cultivar to Cd stress (Additional file 4: Table S3, Additional file 1: Figure S1). HSF and HSPs (large or small) play critical roles in plant immunity, growth, defense, and stress responses [50–53]. In rice, Cd-stress induces *HSPs* expression, in turns it inhibits Cd-induced damage in plant cells [54]. It has been reported that overexpression of HSP enhanced abiotic stress tolerance to heat, drought, abscisic acid, salinity and cold in rice [55]. Cai et al. (2017) reported that silencing of *HSFA1a* in tomato plants could block Cd uptake and reduce *HSP* expression, while *HSFA1a* overexpressing promoted *HSP* expression [53]. The unchanged expression of *HSFs* and *HSPs* indicated that “y” cultivar was insensitive to Cd stimulus. Likewise, the unchanged expression of *OsLHC-II* might indicate more stable photosynthesis, growth, and development of “y” cultivar under Cd stimulus. In addition, 6 aquaporin genes (*PIPs*) were expressed at higher levels in “X” than that in “y” cultivar, and down regulated by Cd treatment only in “X”, but not in “y” (Additional file 1: Figure S1 B). Heavy metal can cause water deficit in plants, which greatly affects plant growth and productivity. The expression of *PIPs* is closely related to heavy metal stress, with distinct expression patterns in different plant species [23–27]. Different reports have shown that heavy metals can trigger the closure of aquaporins due to their abilities to react with the S-H group of the protein [56, 57]. In “X” cultivar, the expression of aquaporin genes was repressed by Cd stress, but no significant changes in “y” cultivar. The decreased expression of aquaporin genes triggered by heavy metals was similar to what Kholodova et al. (2011) reported [25]. The decreased activities of aquaporins lead to low transpiration rate, under which the essential mass apoplastic water flow, determined mainly by the rate of transpiration, was replaced by predominantly cell-to-cell symplastic transport [58], which is regulated at the level of membrane water channels. Our results also indicated that there were significant differences between the two rice cultivars in response to Cd stress.

### Interaction relationship of miRNAs and mRNA in response to cd stress

Among all the DEmiRNAs related to Cd accumulation, we found *osa-miR408-3p, osa-miR528-3p osa-miR528-5p* were commonly up-regulated by Cd stress in both nodes of the two cultivars. The effect of *osa-miR528-3p* has not been studied till now. Both *osa-miR528* and *osa-miR408* family members were differentially expressed in response to abiotic stresses, such as drought [59], low temperature [60], heavy metal [61] and plant defense responses (the reference is about drought) [62]. Cheah et al. (2015) showed that several miRNAs including *osa-miR398*, *osa-miR397*, *osa-miR408-5p* and *osa-miR528-5p* were up-regulated in the drought-susceptible rice variety [59]. This was also true for in cool-tolerant rice (Hitomebore) under cool-temperature [60]. The interaction network showed that 4 transcription factors (TFs), *OsbZIP18*, *OsbZIP23*, *OsMYB5P* and *OsERF141,* were potential target genes of aforementioned miRNAs. Interestingly, osa-miR5493 also showed a negative relationship with OS06G0253100 (*OsHsp20*), OS02G0203000 (*OsbZIP18*) and OS02G0638650 (*OsERF141*) only in *Xiangwanxian No.12*. This implied that osa-miR5493 might also be a key regulator in response to Cd stress via regulating the expression of TFs. As we know, WRKY, bZIP, ERP and MYB proteins play an important role in controlling the expression of their downstream genes in response to Cd stress [63]. The down regulation of Os*ERFs* and Os*bZIPs* was also found in another rice variety after Cd stress [64]. Expression of osa-miR5493 were up-regulated after Cd treatment in “X”. Correspondingly, the unchanged expression of osa-miR5493 and these four TFs further demonstrated that “y” cultivar was insensitive to Cd stress. Consequently, we speculate that osa-miR5493 was an important miRNA in regulating the expression of Os*ERFs* and Os*bZIPs.* SnRKs play an important role in plant biotic interaction in *Arabidopsis thaliana*. SnRK1 overexpression plants displayed enhanced resistance to geminivirus, while *SnRK1* silenced plants were more susceptible than the wild-type plants [65, 66]. The higher expression of *OsSnRK1* in the panicle node suggests that panicle node is the key part of “X” cultivar in response to Cd stress. In addition, *SnRKs* are the upstream regulatory genes of *HSPs* in ABA signal transduction pathway [67, 68]. Therefore, the expression changes of *HSPs* in panicle node of “X” cultivar may be due to the action of *OsSnRK1* and osa-miR5493.

## Conclusions

In this study, we have demonstrated that distinct Cd accumulation in the panicle node and node I in two different rice cultivars was mediated by different gene expression pattern. Both panicle node and node I of “X” cultivar played a key role in blocking the upward transportation of Cd, while only node I played a critical role in “y” cultivar. We have identified a cluster of candidate genes which might be responsible for Cd accumulation in panicle node and node I. Most of these genes (*OsIRT1*, *OsNramp5, OsVIT2*, *OsNRT1.5A, and OsABCC1*) are related to the “transporter activity”. The concerted action of these transporters could block the transport of Cd up to panicle and accumulation in the grains of low Cd-accumulation cultivar. Among the DEGs related to “response to stimulus”, we identified *OsHSP70* and *OsHSFA2d/B2c* down regulated by Cd in “X”, but not in “y” cultivar. MiRNAs including osa-miR528, osa-miR408 and osa-miR5493 family members which were up-regulated by Cd, showed potential roles in lowering Cd accumulation via regulating genes like *bZIP*, *ERF*, *MYB*, *SnRK1* and *HSPs*. The differential gene expression is likely responsible for the low Cd accumulation in “X” cultivar. These findings have provided novel insights into breeding new rice varieties with low Cd accumulation.

## Methods

### Plant materials and treatment

The seeds of two Chinese rice (*O. sativa*) cultivars “Xiangwanxian No. 12” (low Cd accumulation, “X” for short) and “Yuzhenxiang” (high Cd accumulation, “y” for short) were obtained from the germplasm recourses bank of Hunan rice research institute. All seeds were sterilized with prochloraz, soaked in deionized water at room temperature (RT) for 48 h, and germinated at 30 °C for 24 h, then sowed into the fields with normal standard conditions (15–22 °C for nighttime and 20–25 °C for daytime, without Cd stress) on 30th June in 2017. Seedlings with three true leaves were transplanted into pots (54 cm × 41 cm × 23 cm) with the density of 8 plants/4 holes/pot. Rice cultivars were sowed into the experimental pots full of muddy water without (control, “C” for short) or with CdCl_2_·2.5H_2_O (15 mg/Kg; treatment, “T” for short, last for about 4 months) [69]. Each cultivar was planted in 20 replications. Plants were managed with standard fertilization (compound fertilizer N:P_2_O_5_:K_2_O 15:8:12, 3 g/pot) was applied on July 5, 2017; urea was applied on August 16, 2017, 0.6 g/pot) The panicle node (“P” for short) and node I (“N” for short) were collected at grain-filling stage on November 9, 2017. Stem nodes (about 0.3 cm length) were sampled from fresh, leafless stems and snap-frozen in liquid nitrogen. According to the treatment strategies and sampling positions, the group samples were named as XPC, XPT, yPC, yPT, XNC, XNT, yNC and yNT, where “X” notes “Xiangwanxian No. 12”, “y” notes “Yuzhenxiang”, “P” indicates panicle node, “N” indicates node I, “C” represents control and “T” represents Cd treatment. Liquid nitrogen flash frozen samples were stored at − 80 °C before the extraction of RNA. Each sample was pooled by tissues from 3 to 5 individuals, and three repetitive samples were prepared for RNA isolation.

### Determination of cd concentration

The accumulation of Cd in rice “P” and “N” tissues and grain were determined by atomic absorption spectrophotometer (PerkinElmer PinAAcle 900 T, USA). Tissues were drying in a drying box (105 °C for 30 min and at 80°Cto constant weight). 50 mg powder samples were immersed into 1 mL HNO_3_ and digested to transparent solutions. Cooled samples were then diluted into water to a final volume of 13.5 mL. Standard Cd solution was used as quality control samples. Besides, the expression of *OsMAPK, OsHMA3, OsZIP4* and *OsPCS* were detected by qRT-PCR method.

### RNA extraction and libraries construction

Total RNA was extracted from collected samples from two cultivars using TRIzol (Invitrogen, USA). The purification, qualification and quantification were conducted (DNase I, Invitrogen) by Agilent 2100 Bioanalyzer (Agilent Technologies, USA). For the preparation of the miRNA-seq, total RNA fragmentation (16–30 nt) was performed using fragmentation buffer (Ambion, USA), followed with purification, enrichment, ligation (with 3′ and 5′ RNA adapters) and PCR amplification. The finally purified amplification products were regarded small RNA libraries (*n* = 24) used for miRNA-seq. For the preparation of the mRNA-seq, total RNA was reverse transcribed to the first strand cDNA using SuperScript III reverse transcriptase (Invitrogen) with 6-base random primers. The DNA samples were used for the mRNA-seq library (*n* = 24) construction following the instruction from the mRNA-Seq Sample Preparation Kit (Illumina, USA). Illumina HiSeq 4000 sequencing platforms (pair-end 2 × 150 bp for mRNA-seq, and single-end 50 bp for miRNA-seq) were used for the sequencing analysis.

### Data processing

The raw sequencing data in the format of FastQ were quality-controlled using the FastQC (version 0.11.5, http://www.bioinformatics.babraham.ac.uk/projects/fastqc/) by removing the low quality reads and adaptor reads.

### mRNA profiling

The clean reads assembled and aligned to the reference genome sequence (http://rice.plantbiology.msu.edu/pub/data/Eukaryotic_Projects/o_sativa/; IRGSP-1.0.28) was conducted using hisat2 [70]. The reads numbers were counted by htseq-count [71]. Cufflinks (version 2.2.1) [72] was used for the quantitative analysis of the reads by calculating the FPKM values (expected number of Fragments Per kb per Millions reads) of reads in each sample. Principal component analysis (PCA) and sample-to-sample clustering analysis were performed based on the FPKM of reads. The DEGs were identified using DESeq (http://bioconductor.org/packages/release/bioc/html/DESeq.html) [73] by pairwise comparison. DEGs were identified using the Negative binomial distribution test with the criteria of *p* value < 0.05 and |log_2_(Fold Change, FC) | ≥ 1. Up- and down-regulated DEGs were identified as log_2_FC > 1 and log_2_FC < − 1, respectively. The expression profiles of DEGs were presented using hierarchical clustering by pheatmap (version 1.0.10; https://cran.r-project.org/web/packages/pheatmap/index.html).

### miRNA profile analysis

The clean reads generated by miRNA-seq were aligned with the reference genome sequence (*O. sativa*; http://www.mirbase.org/cgi-bin/mirna_summary.pl?org=osa). Small RNA annotation and assignment (rRNA, snRNA, snoRNA and tRNA) were conducted in Rfam [74], cDNA sequencing, species repeat library [75] and miRBase [76, 77]. Small RNA sequences (> 26 nt) were removed using Bowtie [78]. Sequences in the length of 15–26 nt and those unable to match the mRNA transcripts were used for the identification of known and novel miRNAs after removing the repetitive sequences. Novel miRNAs were identified using Mirdeep2 software [79] and RNAfold [80]. The expression of miRNAs were calculated using transcript per million (TPM). PCA and sample-to-sample clustering analyses were performed based on the expression levels. Differentially expressed miRNAs (DEmiRNAs) in response to Cd stimulus were identified using DESeq [73] by pairwise comparison methods, with the threshold of *p* value < 0.05 and |log2(Fold change, FC)| ≥ 0.5. Subsequently, the predictive mRNAs of DEmiRNAs were identified using targetfinder [81]. The overlapping DEGs between DEmiRNAs’ targets were used for further enrichment analysis.

### Enrichment analysis

The DEGs and overlapping DEGs between DEmiRNAs’ targets were separately subjected to the enrichment analysis of Gene Ontoloy (GO; http://www.Geneontology.org/) and KEGG (Kyoto Encyclopedia of Genes and Genomes) pathways [82]. GO categories (biological processes, BP; molecular functions, MFs; and cellular components, CCs) and KEGG pathways related to the DEGs were identified with the criterion of *p* < 0.05. The miRNA-gene regulatory network was constructed using Cytoscape (version 2.8) [83].

### qRT-PCR verification of RNA-seq data

qRT-PCR analysis was performed on a Light Cycler system (Roche) using a SYBR Green PCR Kit (Qiagen). PCR amplification was performed under the following conditions: 94 °C for 5 min, followed by 40 cycles of 94 °C for 15 s, 58 °C for 15 s, and 72 °C for 20 s and a final extension at 72 °C for 5 min. Quantification of gene expression was performed by the comparative 2^-ΔΔC^T method. The validation analysis was performed with three independent biological replicates. The gene-specific primers for qRT-PCR were designed using Primer Premier 5.0 (http://www.PremierBiosoft.com) and were synthesized by Invitrogen (Carlsbad, USA). The correlation analysis of qRT-PCR and RNA-seq were based on Pearson’s correlation coefficient. The primer sequence information were listed in Additional file 6: Table S5.

### Statistical analysis

All experimental data were expressed as mean ± SD. Statistical analysis of all data was performed using the GraphPad Prism 6. Differences were analyzed using the unpaired t-test or one-way ANOVA. *P* < 0.05 and *p* < 0.01 was considered as significant and very significant difference, respectively.

## Supplementary information

**Additional file 1: Figure S1.** The expression level of candidate genes related to Cd transport. T, Cd-treatment; C, control; N, node I; P, panicle node; X, *Xiangwanxian No. 12*; y, *Yuzhenxiang*.

**Additional file 2: Table S1.** Statistics of the differentially expressed genes (DEGs) and miRNAs (DEmiRNAs) by different pairwise comparison.

**Additional file 3: Table S2.** The expression profiles of the 84 common differentially expressed genes associated with “transporter activity” in Fig. 4b.

**Additional file 4: Table S3.** The list of 74 common differentially expressed genes related to “response to stimulus” in Fig. 4c.

**Additional file 5: Table S4.** The list of the differentially expressed miRNAs.

**Additional file 6: Table S5.** The sequences of primers of genes in qRT-PCR.

## Data Availability

The datasets for this study can be found in the BIG SUB database (https://bigd.big.ac.cn/gsub/) with the access No. CRA001894 and CRA001895.

## Abbreviations

“N”: Node I
“P”: Panicle node
“X”: Xiangwanxian No. 12
“y”: Yuzhenxiang
BP: Biological processes
C: Control
CC: Cellular components
Cd: Cadmium
DEGs: Differentially expressed genes
GO: Gene Ontoloy
KEGG: Kyoto Encyclopedia of Genes and Genomes
MF: Molecular functions
NGS: Next-generation sequencing
PCA: Principle component analysis
T: Cd treatment
TFs: Transcription factors
TPM: Transcript per million
