# Supplementary figures and images for: Transcriptome analysis reveals the roles of stem nodes in cadmium transport to rice grain
Source: BMC Genomics. 2020 Feb 6;21:127. doi: 10.1186/s12864-020-6474-7 (PMC7003353; doi:10.1186/s12864-020-6474-7)

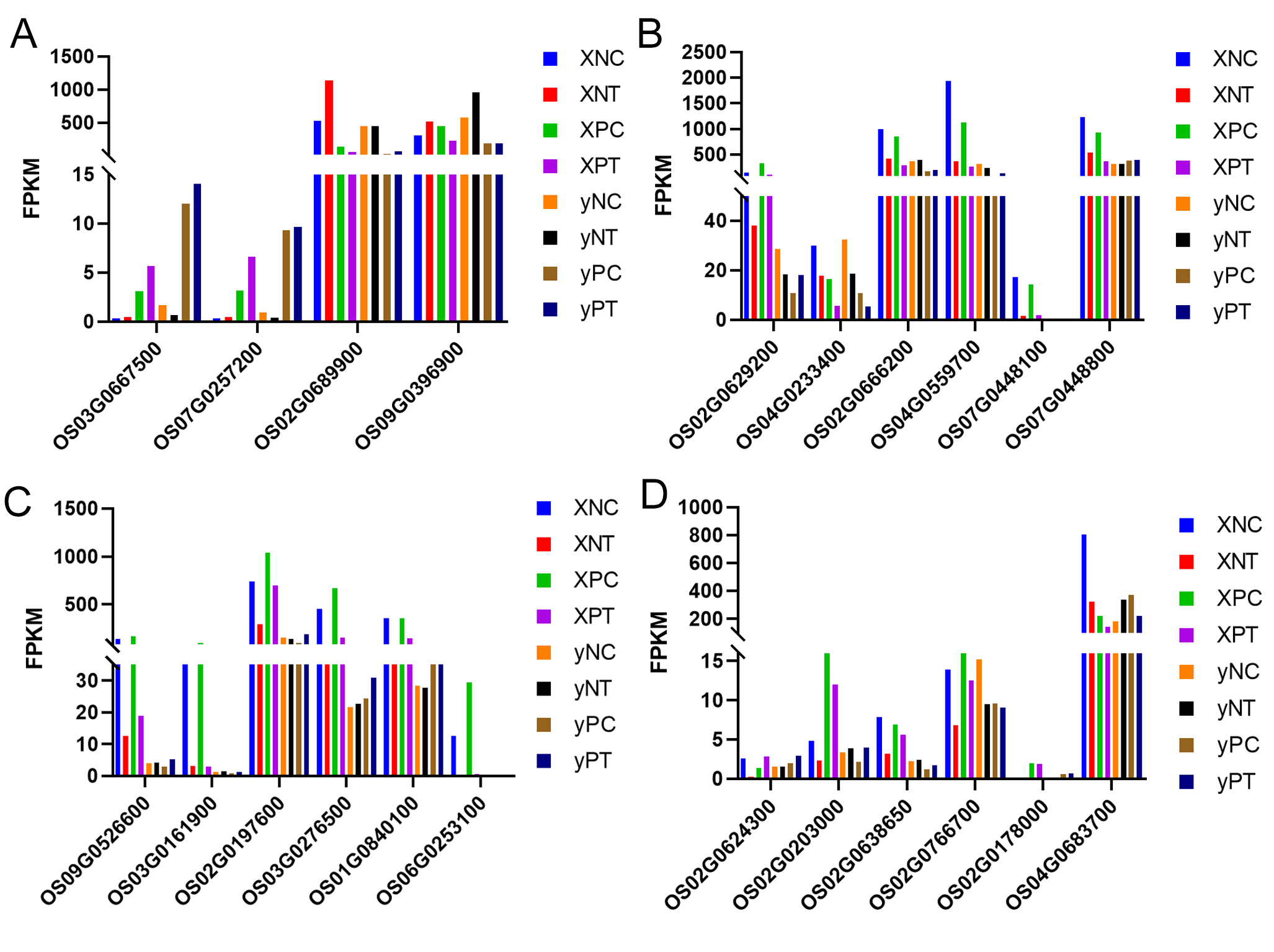

Supplement: Supplementary file 1 — Additional file 1: Figure S1. The expression level of candidate genes related to Cd transport. T, Cd-treatment; C, control; N, node I; P, panicle node; X, Xiangwanxian No. 12; y, Yuzhenxiang. [file 12864_2020_6474_MOESM1_ESM.tif]
