# Supplementary material for: Transcriptome analysis reveals the roles of stem nodes in cadmium transport to rice grain
Source: BMC Genomics. 2020 Feb 6;21:127. doi: 10.1186/s12864-020-6474-7 (PMC7003353; doi:10.1186/s12864-020-6474-7)
Supplement: Supplementary file 2 — Additional file 2: Table S1. Statistics of the differentially expressed genes (DEGs) and miRNAs (DEmiRNAs) by different pairwise comparison. [file 12864_2020_6474_MOESM2_ESM.docx]

**Table S1. The summary of the mRNA-seq and miRNA-seq data.**

| Sample ID | mRNA-seq | | miRNA-seq | | |  |  |  |
| --- | --- | --- | --- | --- | --- | --- | --- | --- |
|  | Clean reads (M) | Map rate (%) | Clean reads (M) | Clean reads Uniq (M) | Map rate (%) | Align in  miRBase (%) | Novel  miRNA cat. | Known  miRNA cat. |
| XPC-1 | 46.82 | 90.72 | 14.72 | 6.32 | 69.20 | 1.54 | 68 | 278 |
| XPC-2 | 46.36 | 90.69 | 12.27 | 4.81 | 70.86 | 1.55 | 62 | 249 |
| XPC-3 | 46.09 | 76.45 | 16.38 | 5.90 | 66.51 | 1.42 | 63 | 275 |
| XPT-1 | 46.59 | 90.78 | 16.53 | 5.42 | 70.73 | 1.60 | 59 | 269 |
| XPT-2 | 46.14 | 89.87 | 15.74 | 4.51 | 68.46 | 1.56 | 59 | 259 |
| XPT-3 | 47.24 | 90.37 | 13.35 | 4.22 | 73.03 | 1.68 | 59 | 266 |
| XNC-1 | 46.09 | 89.92 | 13.87 | 4.98 | 85.45 | 2.27 | 58 | 266 |
| XNC-2 | 46.37 | 88.09 | 17.32 | 6.10 | 86.46 | 1.68 | 62 | 278 |
| XNC-3 | 46.67 | 91.24 | 14.43 | 4.98 | 86.75 | 1.43 | 61 | 254 |
| XNT-1 | 46.35 | 91.71 | 12.54 | 5.01 | 87.77 | 1.32 | 59 | 267 |
| XNT-2 | 45.14 | 92.37 | 14.48 | 5.60 | 89.41 | 1.02 | 59 | 266 |
| XNT-3 | 46.52 | 90.89 | 15.31 | 5.86 | 86.42 | 1.17 | 62 | 274 |
| yPC-1 | 46.21 | 90.29 | 15.14 | 3.91 | 80.85 | 1.66 | 59 | 275 |
| yPC-2 | 46.54 | 90.03 | 12.24 | 2.44 | 82.16 | 1.93 | 55 | 253 |
| yPC-3 | 46.65 | 89.66 | 11.65 | 2.46 | 81.31 | 1.98 | 55 | 256 |
| yPT-1 | 46.79 | 90.7 | 14.10 | 3.25 | 77.44 | 2.09 | 57 | 278 |
| yPT-2 | 45.84 | 90.68 | 16.26 | 3.24 | 76.49 | 1.95 | 56 | 262 |
| yPT-3 | 45.8 | 89.85 | 15.08 | 2.47 | 78.09 | 2.37 | 55 | 249 |
| yNC-1 | 45.78 | 91.13 | 15.59 | 4.86 | 90.17 | 1.33 | 59 | 269 |
| yNC-2 | 46.51 | 90.44 | 14.75 | 4.79 | 92.76 | 0.74 | 57 | 276 |
| yNC-3 | 45.74 | 90.44 | 13.87 | 4.71 | 91.83 | 1.00 | 54 | 274 |
| yNT-1 | 45.86 | 90.3 | 15.64 | 5.54 | 91.42 | 1.10 | 59 | 292 |
| yNT-2 | 46.12 | 90.57 | 14.92 | 5.47 | 92.20 | 0.85 | 60 | 274 |
| yNT-3 | 47.12 | 87.97 | 14.63 | 5.72 | 92.42 | 0.62 | 57 | 283 |
| Total/Average | 1111.34 | 89.79833 | 350.83 | 112.57 | 82.01 | 1.49 |  |  |
